# Retinal and optic nerve degeneration in α-mannosidosis

**DOI:** 10.1186/s13023-018-0829-z

**Published:** 2018-06-01

**Authors:** Juliane Matlach, Thea Zindel, Yasmina Amraoui, Laila Arash-Kaps, Julia B. Hennermann, Susanne Pitz

**Affiliations:** 1 Department of Ophthalmology, University Medical Center, Johannes Gutenberg University Mainz, Mainz, Germany; 2 Department of Pediatrics, University Medical Center, Johannes Gutenberg University Mainz, Mainz, Germany; 3 Orbital Center, Ophthalmic Clinic, Bürgerhospital, Frankfurt, Germany

**Keywords:** α-mannosidosis, Retinal degeneration, Optic nerve atrophy, Ocular findings, OCT

## Abstract

**Background:**

α-mannosidosis is a rare, autosomal-recessive, lysosomal storage disease caused by a deficient activity of α-mannosidase. Typical symptoms include intellectual, motor and hearing impairment, facial coarsening, and musculoskeletal abnormalities. Ocular pathologies reported previously were mainly opacities of the cornea and lens, strabismus, and ocular motility disorders. However, retinal and optic nerve degeneration have been rarely described.

**Methods:**

We report ocular findings of 32 patients with α-mannosidosis. We particularly concentrated on retinal abnormalities which we supported by posterior segment examination, fundus photography, and Spectral-Domain optical coherence tomography (SD-OCT) imaging.

**Results:**

Tapeto-retinal degeneration with bone spicule formations in the peripheral retina or macular changes were seen in three patients (9.4%) on funduscopy; of these, two with optic nerve atrophy. Eight retinal images could be obtained by OCT or fundus photography; of these, six showed thinning of the outer retinal layers on OCT. Overall, optic nerve atrophy was seen in six patients (18.8%); of these, four with partial atrophy. Two patients had partial optic nerve atrophy with no retinal abnormalities on funduscopy. Cataract was seen in two (6.3%), corneal haze also in two patients (6.3%). Six patients (18.8%) had manifest strabismus, four (12.5%) nystagmus, and in five patients (15.6%) impaired smooth pursuit eye movements were seen.

**Conclusion:**

Ocular pathologies are not exclusively confined to opacities of the cornea and lens or strabismus and ocular motility disorders but tapeto-retinal degeneration and optic nerve atrophy may be a common feature in α-mannosidosis. OCT technology helps detecting early outer retinal thinning which can progress with age and potentially leads to vision loss over time.

## Background

α-mannosidosis is a rare, autosomal-recessive, lysosomal storage disease arising from a deficiency in lysosomal α-mannosidase caused by mutations in *MAN2B1* located on chromosome 19 with an estimated prevalence of 0.5–1:500.000. Deficiency of α-mannosidase leads to accumulation of mannose-rich oligosaccharides in all tissues resulting in cell dysfunction. Clinical characteristics show different phenotypic variations including cognitive disability with gradual impairment of speech and mental functions, psychosis, motor function and hearing disturbances, facial and skeletal abnormalities, and immune deficiency. Two α-mannosidosis phenotypes have been described based on clinical severity: a severe infantile form (type I) characterized by early death due to rapid progressive central nervous system involvement and a milder phenotype with a slower disease progression and survival into adulthood (type II) [1, 2]. Diagnosis of α-mannosidosis is made by measuring acid α-mannosidase activity in leukocytes or other nucleated cells and can be confirmed by genetic testing. Elevated urinary secretion of mannose-rich oligosaccharides is suggestive of the disease, however not diagnostic [1]. Öckerman first described a Hurler-like appearance of a four-year-old boy in 1967. In a postmortal examination, a large amount of oligosaccharides, especially mannose-rich oligosaccharides, was found in his organs and tissues [3]. He therefore named the disorder “mannosidosis” [4]. As it is a deficiency in lysosomal α-mannosidase, oligosaccharides accumulate in different tissues and organs. Since these early clinical descriptions of mannosidosis, many research groups have contributed to the characterization of the presenting phenotype, enzyme and corresponding genes in several species including ocular pathologies in 20 α-mannosidosis patients [5–8]. Early reports have described strabismus, opacities of the cornea and lens as typical ocular symptoms and pathologies in mannosidosis [5]. However, only recently, retinal abnormalities and optic nerve atrophy have been described in a few case reports and confirmed by electrophysiology or optical coherence tomography (OCT) as fundus biomicroscopy only reveals subtle retinal changes [9, 10]. Currently, there are two treatment options for α-mannosidosis: stem cell transplantation and enzyme replacement therapy (ERT). Haematopoietic stem cell transplantation has been reported in less than 20 patients with different outcomes and a high risk of morbidity and mortality [11]. The efficacy and safety of ERT with a recombinant human α-mannosidase (velmanase alfa) has been studied in randomized clinical trials [12] and let to approval by the European Medicines Agency (EMA) in January 2018.

Here, we report ocular findings of 32 patients including a report of two siblings with confirmed α-mannosidosis and especially concentrated on retinal degeneration which we supported by posterior segment examination, fundus photography and Spectral-Domain OCT (SD-OCT).

## Methods

In total, 32 patients with α-mannosidosis were examined; of these 25 participated in a multicenter, multinational prospective natural history study of α-mannosidosis (Trial-ID: rhLAMAN-01). Clinical evaluations included physical examination, recording of medical history, measurement of endurance by six-minute walk test and three-minute stair climb-test, lung function testing, hearing test, echocardiography and electrocardiography, and laboratory testing. These results were previously published by Beck et al. [13]. In this report, general ophthalmic investigations such as vision, anterior and posterior segment abnormalities were only briefly described. These 25 patients were initially examined between 2007 and 2009 and followed-up over two to three years. In addition to this, we examined seven more patients with α-mannosidosis between 2008 and 2017.

In total, seven patients received ERT with velmanase alfa; of these, six (Man-1, Man-3, Man-4, Man-21, Man-28, Man-31) began treatment during or before our ophthalmic examinations; only Man-32 received his treatment after examination (Tables 1 and 2).

**Table 1:** Demographics and ocular findings of all patients

n of patients	32
Sex, male/female	19 (59.4%)/13 (40.6%)
Age, years	
1st presentation	18.4 ± 11.8 (1–53)
Last presentation	20.8 ± 11.6 (3–53)
BCVA, decimal	
1st presentation	0.56 ± 0.28 (0.04–1.00)
Last presentation	0.60 ± 0.25 (0.10–1.00)
ERT with velmanase alfa	7 (21.9%)
Ocular pathologies^a^	
Lens opacity	3 (9.4%)
Corneal haze	2 (6.3%)
Optic nerve atrophy, also partial	6 (18.8%)
Tapeto-retinal degeneration (by funduscopy)	3 (9.4%)
**Tapeto-retinal degeneration (by OCT)**	**6/8** ^b^
**Tapeto-retinal degeneration (by photo)**	**3/8** ^b^
Manifest strabismus	6 (18.8%)
Nystagmus	4 (12.5%)
Saccadic/hypometric eye movements	5 (15.6%)

Data are absolute vales (%), mean ± standard deviation (min-max), as appropriate.

^a^ocular pathologies that were noticed at all presentations

^b^8 retinal images could be obtained by OCT/fundus photography; of these, 6 showed retinal degeneration on OCT (thinning of the outer layers); in 1 woman, macular edema was seen due to tapeto-retinal degeneration.

Abbreviations: *n* number of patients, *BCVA* best-corrected visual acuity, *ERT* enzyme replacement therapy, *OCT* optical coherence tomography

**Table 2:** Genotype and ocular characteristics of all patients

					*Ophthalmic examination* ^c^						
*pat.no., gender*	*label cDNA* *(allele 1/ allele 2)*	*label protein* *(allele 1/ allele 2)*	*ERT* ^*a*^	*age* ^*b*^ *[yrs]*	*BCVA right/left*	*anterior segment*	*posterior segment*	*optic nerve*	*strabismus/nystagmus/motility*	*imaging*	*macular OCT*
Man-1, male	c.418C > T/c.418C > T	p.Arg140X/p.Arg140X	yes, from the age of 14 yrs. ongoing	18	0.8/0.7	normal	normal	normal	unremarkable	OCT + photo	thinning of outer retinal layers
Man-2, male	c.1830 + 1G > A/c.2248C > T	p.?/ p.Arg750Trp	no	17	0.63/0.63	normal	normal	normal	unremarkable	no	n.a.
Man-3, male	c.1358C > T/c.1358C > T	p.Ser453Phe/p.Ser453Phe	yes, from the age of 15 yrs. ongoing	21	0.5/0.63	normal	normal	partial atrophy	saccadic motility	OCT + photo	thinning of outer retinal layers
Man-4, female	c.1358C > T/c.1358C > T	p.Ser453Phe/p.Ser453Phe	yes, from the age of 22 yrs. ongoing	25	0.25/0.4	normal	normal	partial atrophy	saccadic motility	OCT + photo	thinning of outer retina, macular edema
Man-5, male	c.164G > T/c.599A > T	p.Cys55Phe/p.His200Leu	no	25	0.63/0.8	normal	normal	normal	unremarkable	no	n.a.
Man-6, male	c.164G > T/c.599A > T	p.Cys55Phe/p.His200Leu	no	19	0.5/0.63	normal	normal	normal	unremarkable	no	n.a.
Man-7, female	c.2248C > T/c.2248C > T	p.Arg750Trp/p.Arg750Trp	no	26	0.32/0.5	cerulean cataract	normal	normal	unremarkable	no	n.a.
Man-8, female	c.1830 + 1G > C /c.1830 + 1G > C	p.VAL549_Glu610del/ p.VAL549_Glu610del	no	34	0.32/0.32	normal	normal	normal	down-beat nystagmus, saccadic motility	no	n.a.
Man-9, male	c.2248C > T/c.2248C > T	p.Arg750Trp/p.Arg750Trp	no	29	n.a.	normal	tapeto-retinal, one spicules	atrophy	exotropia, down-beat nystagmus	photo	n.a.
Man-10, male	c.484_487dupGCCA/c.484_487dupGCCA	p.Thr163Serfsx25/p.Thr163Serfsx25	no, but stem cell transplantation at the age of 3 yrs	3	0.5/0.25	normal	normal	normal	unremarkable	no	n.a.
Man-11, male	c.338-348dup11/c.338-348dup11	p.Ile117profsX44/p.Ile117profsX44	no	3	binocular 0.8	normal	normal	normal	unremarkable	no	n.a.
Man-12, male	c.2921_2922delCA/c.2921_2922delCA	p.(Thr974ArgfsTer80/p.(Thr974ArgfsTer80	no	14	binocular 0.1	normal	normal	normal	unremarkable	no	n.a.
Man-13, female	c.1830 + 1G > C/c.2248C > T	p.VAL549_Glu610del/p.Arg750Trp	no	19	1.0/0.63	normal	normal	normal	unremarkable	no	n.a.
Man-14, male	c.844C > T/c.844C > T	p.Pro282Ser/p.Pro282Ser	no	16	0.2/0.2	normal	tapeto-retinal degeneration	normal	unremarkable	no	n.a.
Man-15, female	c.2248C > T/c.2426 T > C	p.Arg750Trp/p.Leu809Pro	no	20	0.5/0.5	normal	normal	normal	unremarkable	no	n.a.
Man-16, male	c.2248C > T/c.2248C > T	p.Arg750Trp/p.Arg750Trp	no	15	0.8/0.63	corneal haze	normal	normal	unremarkable	no	n.a.
Man-17, male	c.2724G > A/c.2724G > A	p.TRP908X	no	5	0.5/0.5	normal	normal	normal	unremarkable	no	n.a.
Man-18, male	c.1351G > T/c.[1501 T > A; 2849G > C]	p.Gly451Cys/p.([Cys501Ser; Arg950Pro])	no	9	1.0/1.0	normal	normal	normal	unremarkable	no	n.a.
Man-19, female	c.2248C > T/c.2248C > T	p.Arg750Trp/p.Arg750Trp	no	33	0.63/0.63	normal	myelinated nerve fibers	partial atrophy	unremarkable	no	n.a.
Man-20, male	c.283G > C/c.283G > C	p.Ala95Pro/p.Ala95Pro	no	13	0.8/0.8	normal	normal	normal	unremarkable	no	n.a.
Man-21, male	c.2234C > G/c.2234C > G	p.Thr745Arg/p.Thr745Arg	yes, from the age of 25 yrs. ongoing	29	1.0/0.9	normal	normal	normal	hypometric motility	OCT + photo	thinning of outer retinal layers
Man-22, female	c.788C > T/c.2355G > A	p.Pro263Leu/p.Arg757MetfsTer6	no	18	n.a.	normal	normal	normal	esotropia	no	n.a.
Man-23, male	c.1816delA/c.1830 + 1G > C	p.Thr606ProfsTer18/p. Val549_Glu610del	no	42	0.9/0.5	normal	normal	normal	esotropia, nystagmus on lateral gaze	no	n.a.
Man-24, female	c.1816delA/c.1830 + 1G > C	p.Thr606ProfsTer18/p. Val549_Glu610del	no	40	0.32/0.8	normal	macular changes	atrophy	esotropia	no	n.a.
Man-25, male	c.1310-2A > G/c.2248C > T	p.?/ p.Arg750Trp	no	27	0.25/0.25	normal	normal	partial atrophy	unremarkable	no	n.a.
Man-26, male	c.283G > C/c.283G > C	p.Ala95Pro/p.Ala95Pro	no	16	1.0/0.8	corneal haze	normal	normal	unremarkable	no	n.a.
Man-27, female	c.1351G > T/c.1830 + 1G > C	p.Gly451Cys/p. Val549_Glu610del	no	27	0.5/0.63	normal	normal	normal	unremarkable	no	n.a.
Man-28, female	c.2248C > T/c.1046insC	p.Arg750Trp/ p.?	yes, from the age of 7 yrs. ongoing	14	1.0/0.8	normal	normal	normal	unremarkable	OCT + photo	normal
Man-29, female	c.263-2A > c/c.1204G > A	p.?/p.Glu402Lys	no	53	0.8/0.7	normal	normal	normal	saccadic motility	OCT + photo	thinning of outer retinal layers
Man-30, female	c.215A > T/c.2471G > A	p.His72Leu/p.Gly824Glu	no	24	0.63/1.0	anterior polar cataract	normal	normal	esotropia, up-beat nystagmus	OCT + photo	thinning of outer retinal layers
Man-31, female	c.2248C > T/c.2248C > T	p.Arg750Trp/p.Arg750Trp	yes, from the age of 3 yrs. ongoing	3	binocular 0.5	normal	normal	normal	unremarkable	no	n.a.
Man-32, male	n.a.	n.a.	yes, from the age of 10 yrs. ongoing	10	0.2/0.63	normal	normal	normal	exotropia	OCT + photo	normal

^a^ERT: weekly treatment with velmanase alfa; ^b^age at the last ophthalmic examination; ^c^ophthalmic findings at the last presentation

Abbreviations: *BCVA* best-corrected visual acuity, *ERT* enzyme replacement therapy, *n.a.* not available/applicable, *OCT* optical coherence tomography, *yrs.* years

### Ophthalmological assessment

Ophthalmic examinations were carried out at the Department of Ophthalmology, University Medical Center Mainz, Germany.

All patients or their relatives were asked for ocular history and medication, and underwent a standard ophthalmic examination including best-corrected visual acuity (BCVA) testing, slit-lamp biomicroscopy for anterior segment and indirect ophthalmoscopy for fundus examination, and assessment of strabismus, ocular motility and nystagmus. Measurement of the optic nerve head, peripapillary retinal nerve fiber layer and macular region using SD-OCT (Spectralis OCT, Heidelberg Engineering GmbH, Heidelberg, Germany) was introduced later as part of our examinations and was therefore only performed in eight of 32 patients. Due to individual patient’s abilities, not all of the mentioned examinations could be performed. Some of these proved to be difficult due to the patients‘ mental and physical disabilities, especially examinations which need patients’ cooperation (e.g. vision and motility testing, photographs, OCT). No visual acuity could be obtained from two patients, one with severe optic nerve atrophy and retinal degeneration. Ocular examinations of four patients were proved to be very difficult to impossible. Our analyses are mainly descriptive and report single cases supported by fundus photographs and retinal imaging by SD-OCT.

## Results

A total of 32 patients with α-mannosidosis were included and ophthalmlogically examined. All patients were assigned to the attenuated form of α-mannosidosis (type II). Table 1 summarizes the patients’ demographics and ocular abnormalities. Some patients were only seen once whereas others were followed-up over many years.

Mean BCVA at first presentation was 20/40 (decimal 0.56 ± 0.28) with a range between 20/500 (decimal 0.04) and 20/20 (decimal 1.00); BCVA at the last (available) presentation was 20/32 (decimal 0.60 ± 0.25) with a range between 20/200 (decimal 0.10) and 20/20 (decimal 1.00). Cataract was seen in two (6.3%) and corneal haze also in two patients (6.3%). Manifest strabismus was seen in six (18.8%), nystagmus in four (12.5%) and impaired smooth pursuit or hypometric saccades in five patients (15.6%) at the first presentation or during follow-up.

### Fundus abnormalities and OCT findings

We have seen retinal changes on funduscopy in some patients during the course of our examinations over years; however, it was only until later that we performed SD-OCT and noticed early changes of the outer retinal layers. We could bring back and examine four patients from the natural history study again and found thinning of outer retinal layers outside the fovea on OCT in all four patients. Of the seven additional patients that were included after the natural history study, an OCT was obtained in four; of these two had retinal degeneration of the outer layers.

**Fig. 1:**
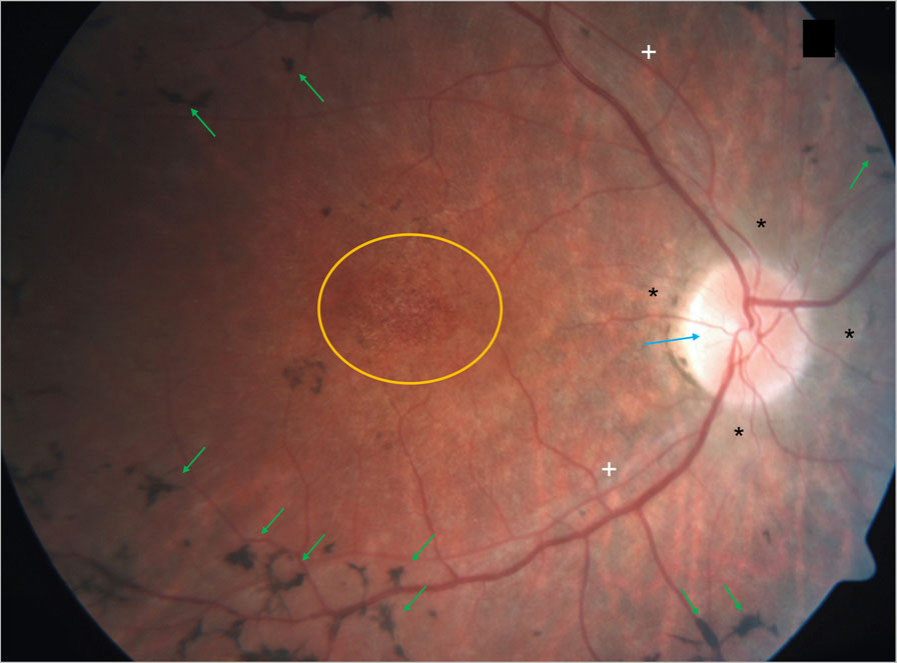
Tapeto-retinal degeneration in α-mannosidosis. Fundus photograph of a 33-year-old man with Retinitis Pigmentosa-like changes in both eyes. Peripheral pigment clumping (**green arrows**), partial optic nerve atrophy (**blue arrow**), chorioretinal atrophy around the optic disc (**black stars**), thin retinal vessels (**white crosses**), and mottled patches in the macula (**yellow circle**) were seen

Overall, optic nerve atrophy was seen in six patients (18.8%); of these four with partial atrophy. Tapeto-retinal degeneration with bone spicule formations in the peripheral retina or macular changes were seen in three patients (9.4%) on funduscopy (Fig. 1); of these, two with optic nerve atrophy. Two patients had partial optic nerve atrophy with no retinal abnormalities on funduscopy. However, the other two patients with partial atrophy only showed thinning of the outer retinal layers on OCT that was not seen on funduscopy.

Table 2 shows the genotype and ocular characteristics of all patients in detail.

### Report of two siblings

#### Case 1

**Fig. 2:**
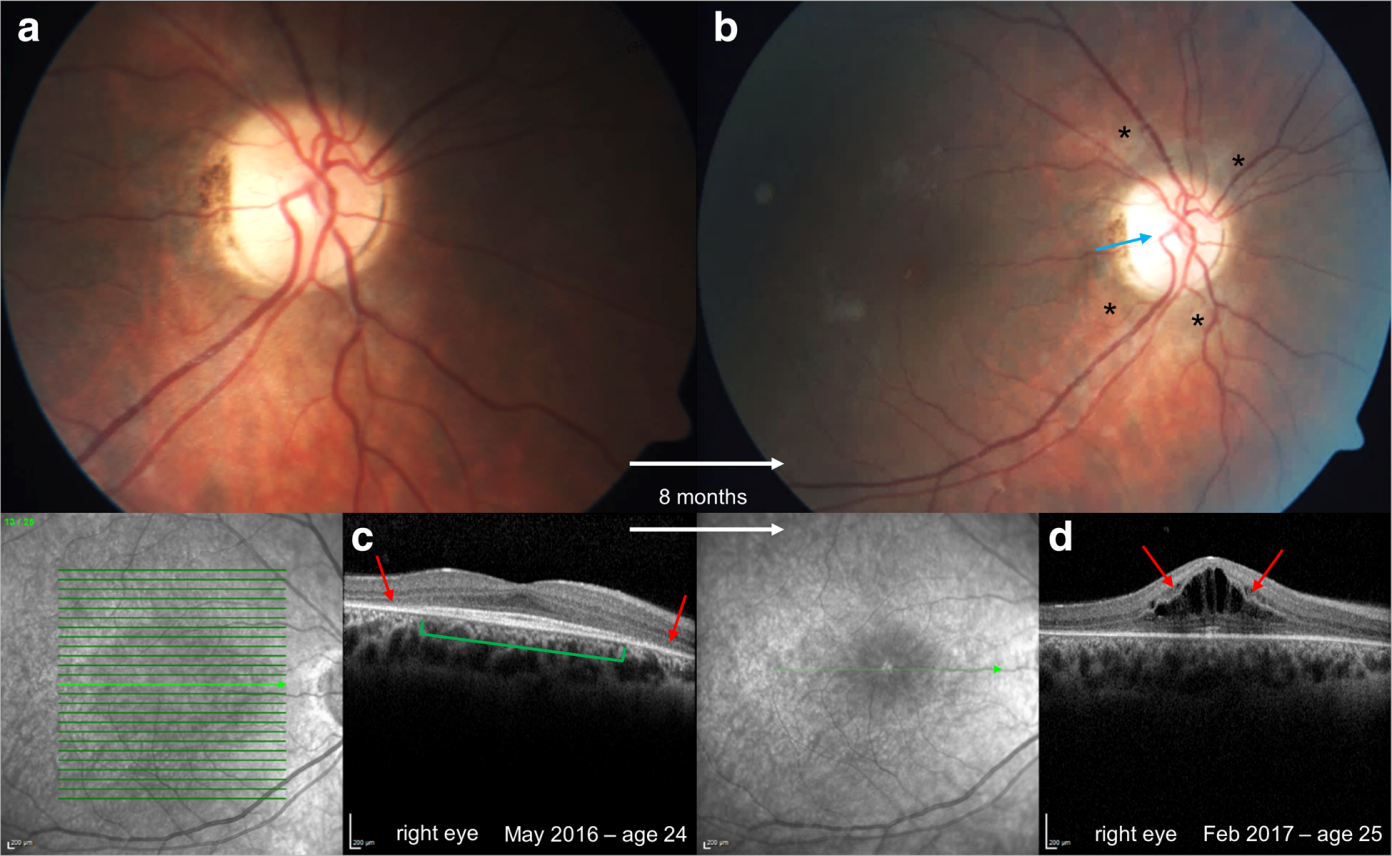
Tapeto-retinal degeneration in α-mannosidosis in two siblings. Fundus photographs (**a, b**) of the sister revealed progression of retinal pigment epithelium (RPE) atrophy outside the macula with yellow-white deposits around the optic disc and chorioretinal atrophy (**black stars**, **b**), and partial optic nerve atrophy (**blue arrow**, **b**). Optical coherence tomography (OCT) showed perifoveal thinning of the outer retinal layers and RPE (**red arrows**, **c**) with normal retinal layers in the fovea (**green bracket**, **c**). A progression of the outer retina thinning was seen and a cystic macula edema has developed within a year’s time at the age of 25 (**red arrows**, **d**)

Case 1 is a girl who was diagnosed at the age of two and a half as having α-mannosidosis due to motor development delay and thoracolumbar kyphosis. At the age of 15 she presented with coarse facial features, severe hearing impairment, intellectual disability, ataxia, mitral valve insufficiency grade I, and recurrent infections. Her BCVA at the age of 15 was 0.63 (20/32) in both eyes without evidence of an afferent pupillary defect, an intraocular pressure reading of 15 mmHg in both eyes and an unremarkable anterior segment without corneal haze or cataract. Fundus examination revealed a normal optic nerve head; the posterior pole was unremarkable with a normal foveal reflex before the start of ERT. At the age of 22, weekly treatment of velmanase alfa was initiated. SD-OCT showed retinal thinning, especially with loss of the outer retinal layers, atrophy of the RPE outside the fovea at the age of 24. Within a year at the age of 25 she developed cystic macular edema seen on SD-OCT with a reduced vision of 0.25 (20/80); the posterior pole demonstrated a partial optic nerve atrophy and mottled nummular yellow to white deposits at the level of the RPE, most notable surrounding the optic nerve head (Fig. 2).

#### Case 2

**Fig. 3:**
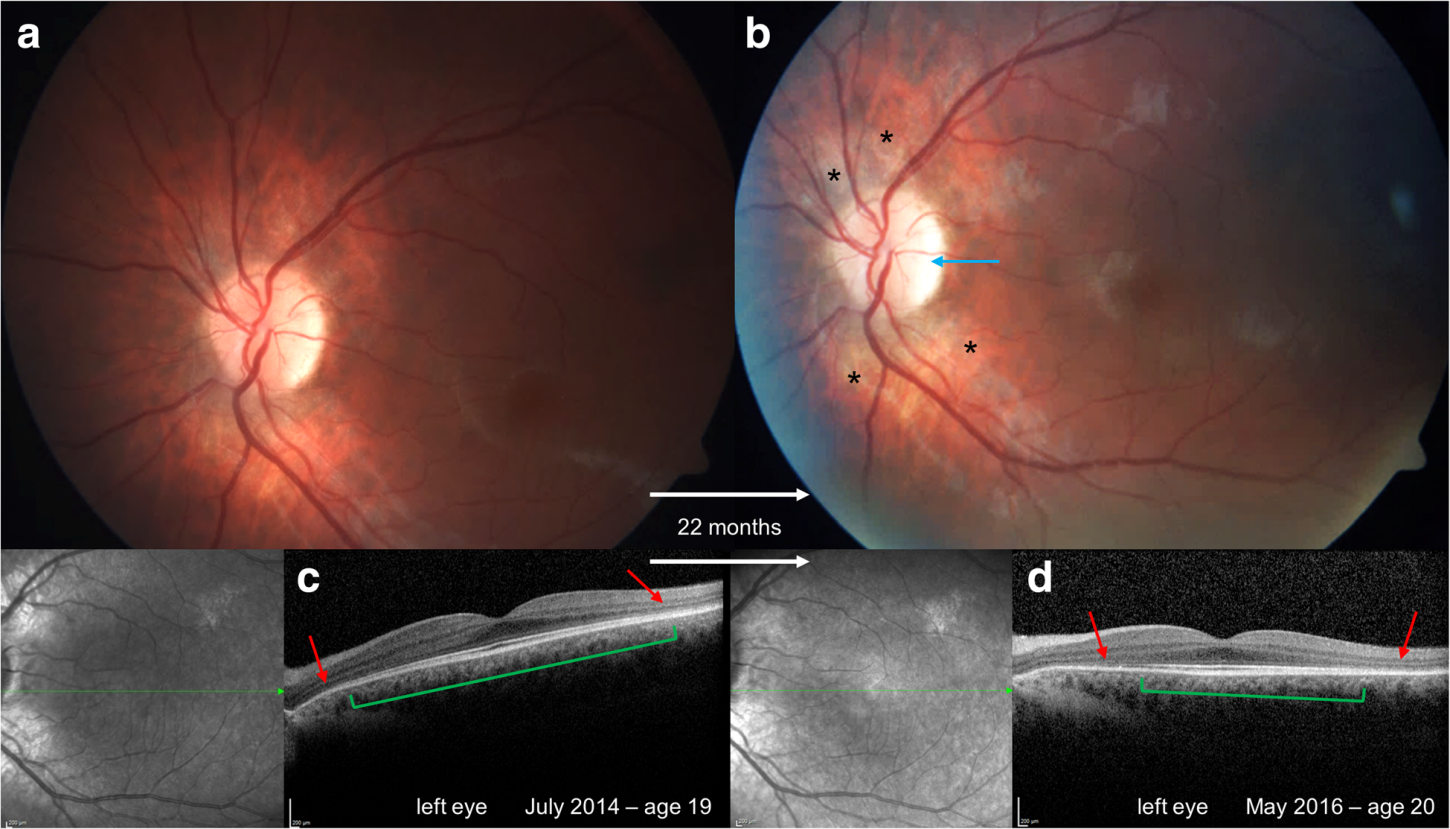
Tapeto-retinal degeneration in α-mannosidosis in two siblings. Fundus photographs (**a, b**) of the brother showed early partial optic nerve atrophy (**blue arrow**, **b**) but without any yellow-white or pigmented deposits, besides a more visible choroid around the optic nerve head (**black stars**, **b**). Optical coherence tomography (OCT) showed a perifoveal thinning of the outer retinal layers and retinal pigment epithelium (RPE, **red arrows**, **c, d**) with normal retinal layers in the fovea (**green bracket**, **c, d**). However, outer retina thinning progressed over time (smaller **green bracket**, **d**) with a larger perifoveal area of outer retinal atrophy (**red arrows**, **d**)

Her brother was diagnosed with α-mannosidosis just after birth. Hearing impairment and motor development delay were the first recognized symptoms. At the age of 12 he presented with various abnormalities including coarse facial features, intellectual disability, ataxia, thoracolumbar kyphosis, aortic valve insufficiency grade I, and recurrent infections. His BCVA was 0.63 (20/32) in both eyes with a normal pupillary reaction on his first examination at the age of 12. Before the start of ERT, anterior and posterior segment appeared unremarkable without corneal haze or cataract, optic nerve atrophy or retinal degeneration. Weekly treatment of velmanase alfa was started at the age of 15. During follow-up at the age of 21, SD-OCT demonstrated early outer retinal thinning without any evidence of yellow-white or pigmented deposits, besides a more visible choroid around the optic nerve head and early partial optic nerve atrophy on funduscopy and fundus photography (Fig. 3).

## Discussion

We report ocular abnormalities in a large patient population of 32 patients with α-mannosidosis. In this to date largest case series investigating ocular manifestations of α-mannosidosis patients we detected a high incidence of retinal degeneration and optic nerve atrophy. Obvious tapeto-retinal characteristics of the retina as detected by funduscopy were rarely found; however early thinning of the outer retina seen on SD-OCT may suggest a progressive nature of this retinal degeneration in α-mannosidosis. Optic nerve atrophy can be associated with retinal degeneration but we have also seen it in some of our patients without any retinal abnormalities. Also of importance is our observation that four of seven patients treated with velmanase alfa developed retinal degeneration despite ERT. Corneal and lenticular opacities as well as strabismus and motility disorders were less frequent in our cohort of α-mannosidosis patients and may be a nonspecific finding as encountered in other storage diseases.

Bennet and co-workers reported on ocular pathologies in two unrelated patients diagnosed with mannosidosis. One had type I mannosidosis affected from early childhood with poor vision, esotropia and cataracts in both eyes. The other was diagnosed with type II mannosidosis in late adulthood and maintained normal vision but deteriorated with progressive neurologic systems and horizontal nystagmus on lateral gaze [6]. In a report by Arbisser et al. three patients with α-mannosidosis showed similar lenticular opacities without any corneal haze. Ophthalmoscopic anomalies were noticed in two younger patients despite normal electrophysiology. [5]. Histological studies of the eye in humans with α-mannosidosis are not available; however Jolly et al. studied this in a bovine model and found vacuoles in different cell types including corneal epithelium, Descemet’s membrane, corneal endothelium, corneal fibroblasts, pigmented cells, lens epithelium, lens fibers, pigment epithelium and also all cell types of the neuroretina. Histological examinations showed that the mannose-rich oligosaccharides were stored in vacuoles. They hypothesized that this may be the cause of lens and corneal opacities in humans with α-mannosidosis [7]. Moreover, the retained storage material in the retina can lead to photoreceptor loss and tapeto-retinal degeneration [10]. This may also be an explanation for the progression of clinical symptoms including retinal degeneration and optic nerve atrophy with age as we have seen in the two siblings during follow-up. In contrast to previously published articles on ocular pathologies in α-mannosidosis that mainly concentrated on opacities of the cornea or lens, strabismus, nystagmus and other motility disorders, Springer and co-workers described late-onset retinal dystrophy characterized by decreasing visual acuity and diminished Ganzfeld electroretinograms in two brothers with type II α-mannosidosis [9]. Both were in their thirties when they were first examined. They had decreasing vision despite normal findings on fundus examination. Electroretinography showed borderline scotopic and photopic responses; however clinical examination was challenging due to the patients’ reduced mental capacity and inability to cooperate [9]. More recently, Courtney and Pennesi published a short report of two cases of retinal dystrophy in α-mannosidosis [10]. This case report is the first to describe, in addition to corneal and lenticular opacity, chorioretinal atrophy with retinal thinning, loss of the outer retina and RPE as well as granular areas of hyper- and hypoautofluorescence in the macula and surrounding the optic nerve using OCT, fundus autofluorescence and fundus photography [10].

Interestingly, certain similar ocular abnormalities can be found in other storage diseases. In mucopolyasaccharidoses (MPS), glycosaminoglycans accumulate in the retina and induce retinal degeneration, pigmentary retinopathy with bony spicules, or depigmented chorioretinopathy similar to our findings in α-mannosidosis [14–16]. In a recent report by Seok et al. four patients with different types of MPS showed retinal degeneration with perifoveal thinning of the outer retinal layers on SD-OCT often despite normal fundus morphology [15]. This is also in line with our findings that SD-OCT displays early degeneration of the retina with no or subtle retina changes on funduscopy.

Another interesting finding of our study is that ERT with velmanase alfa did not protect some of our patients receiving ERT during the observational period from developing retinal degeneration. A phase I-II study evaluated the efficacy and safety of the recombinant human α-mannosidase (velmanase alfa) in 10 patients with weekly therapy over 12 months. Borgwardt et al. showed promising results with improved motor and cognitive function and reduced oligosaccharide concentrations in the serum, urine and cerebrospinal fluid [12]. Ocular changes were not evaluated in this study. In our patients on long-term ERT, only those starting treatment after the age of 14 years developed progressive retinal degeneration. One patient starting treatment at the age of 7 years did not yet develop retinal or optic nerve degeneration. Theoretically, the efficacy of ERT might be better the younger the patients are when starting treatment. However, we cannot conclude yet that ERT might prevent ophthalmological changes in patients with α-mannosidosis even on long-term ERT.

## Conclusions

In conclusion, ocular pathologies in α-mannosidosis do not confine to corneal or lenticular opacities. Our investigations revealed retinal and optic nerve degeneration as common eye pathologies in α-mannosidosis. This is in contrast to some of the earlier reports of ophthalmological findings in α-mannosidosis with no clinical significance to the patients. OCT provides early diagnosis of retinal degeneration by showing thinning of the outer retinal layers when conventional funduscopy or photography fails to detect it. Also optic nerve atrophy may be a new feature of α-mannosidosis. Future larger prospective studies with retinal imaging such as OCT are required to evaluate incidence of retinal degeneration in α-mannosidosis as it may be commonly seen when systematically examined with OCT. Furthermore it has to be investigated whether and when retinal degeneration progresses in α-mannosidosis to a potentially vision-threatening ocular disease and how therapeutic principles such as ERT may influence retinal degeneration.

## Abbreviations

BCVA: Best-corrected visual acuity
EMA: European Medicines Agency
ERT: Enzyme replacement therapy
MPS: Mucopolyasaccharidoses
OCT: Optical coherence tomography
RPE: Retinal pigment epithelium
SD-OCT: Spectral-Domain optical coherence tomography
